# Ectopic Atrial Tachycardia in a 12-Month-Old Girl Treated With Ivabradine and Beta-Blocker, a Case Report

**DOI:** 10.3389/fped.2020.00313

**Published:** 2020-06-16

**Authors:** Holger Michel, Frank Heißenhuber, Sven Wellmann, Michael Melter, Stephan Gerling

**Affiliations:** ^1^University Children's Hospital Regensburg (KUNO), University of Regensburg, Regensburg, Germany; ^2^Clinic for Cardiology-Electrophysiology, Hospital Barmherzige Brüder, Regensburg, Germany

**Keywords:** tachycardia, atrial, Ivabradine, beta-blocker, children, case report

## Abstract

We report on a 12-month-old girl with an ectopic atrial tachycardia successfully treated with the combination of a beta blocking agent and Ivabradine that acts on cardiac pacemaker cells by selectively inhibiting the I_f_ channel. Standard therapy had failed to control the tachycardia before. No side effects attributable to Ivabradine were noticed. Due to its mechanism of action Ivabradine is a promising novel agent for the therapy of tachycardia due to increased automaticity. Reports on the use of Ivabradine in young children or infants are rare, but show promising results for congenital junctional ectopic tachycardia. This report adds the second case of ectopic atrial tachycardia in this age group and novel treatment with Ivabradine to the literature.

## Background

Ectopic atrial tachycardia (EAT) is a known cause of supraventricular tachycardia in children (1). Due to its incessant nature it can lead to a tachycardia induced cardiomyopathy. In younger children it is mostly responsive to antiarrhythmic medication, but a considerable number of patients is not sufficiently treated by a first line regime and needs a combination of two or more antiarrhythmic drugs. As spontaneous resolve is common in this age group, therapy usually should be continued for an average time of 1 year. In older children (≥3 years) failure of antiarrhythmic medication regimes is more common and early radiofrequency ablation is a reasonable approach (2).

Ivabradine lowers the heart rate by selectively inhibiting a mixed sodium-potassium inward current (I_f_) controlling the spontaneous diastolic depolarization in pacemaker cells (3) and was FDA-approved for stable chronic heart failure in adults. With respect to its mechanism of action it is a promising agent for therapy of tachycardia due to increased automaticity. In the pediatric age group there are currently only case reports and a few small case series published. While two case series demonstrated a promising effect with congenital junctional ectopic tachycardia (JET) in infants (4, 5), reports on its application in EAT are limited to older children and adults (6–9). Reports on the use of Ivabradine in infants or/and young children with EAT are scarce (9).

Here, we report the successful use of Ivabradine in combination with a beta blocker for the therapy of EAT in an 1-year old girl.

## Case Report

An one-year old girl presented a persistent tachycardia during a routine visit at the local pediatrician and was immediately transferred to KUNO university hospital. There had been a benign febrile infection 1 week prior to admission. Personal and family history was non-contributory to the tachycardia. Six months before, the previous check at the pediatrician had been uneventful. On admission she had mild signs of heart failure with delayed capillary refill time (3 s) and an enlarged liver. She had a tachycardia with 240 beats/min. Echocardiography showed structural normal anatomy with moderately reduced contractility while tachycardia. Laboratory studies including blood count, inflammatory biomarkers and cardiac troponin were normal but NT-proBNP was significantly elevated (maximum 2,685 pg/ml, lab reference <130 pg/ml).

**Figure 1:**
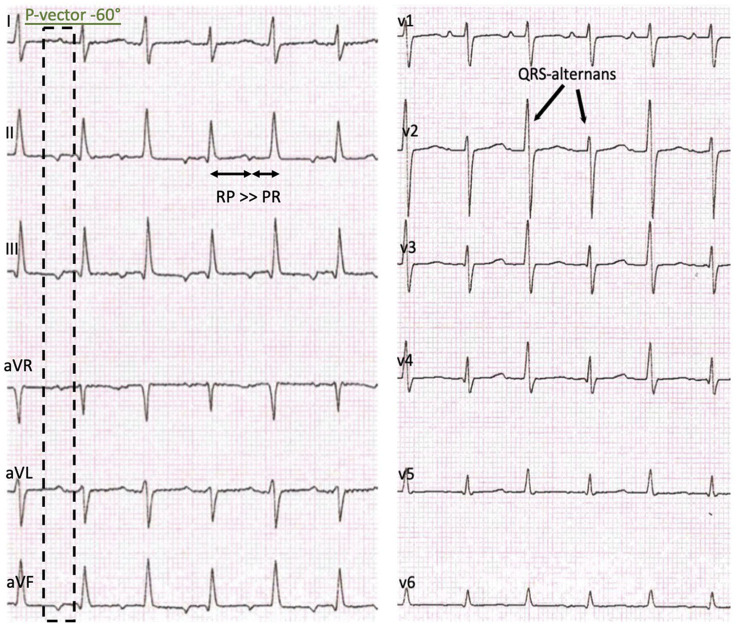
Initial presentation: 12-lead-ECG with a regular sustained small QRS-Complex tachycardia, P-vector −60°, a delay of the atrioventricular conduction, RP > PR and a QRS-complex amplitude alternans.

**Figure 2:**
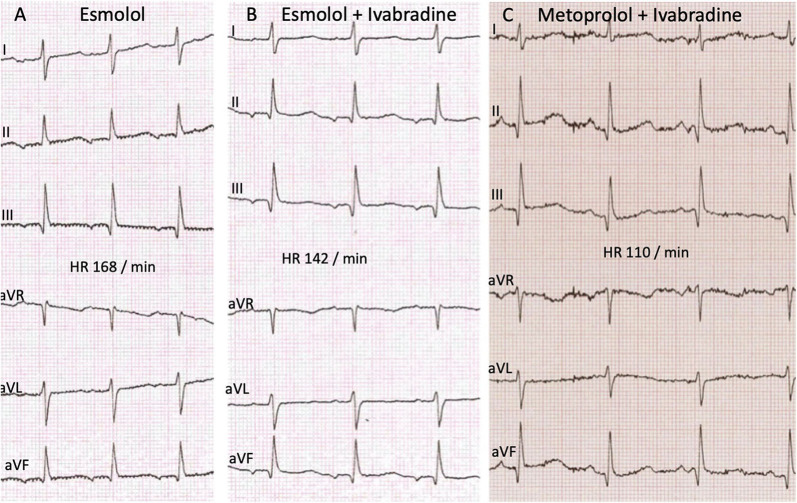
ECG recordings at different time points of the therapy showing no significant changes in the time intervals and de- or repolarisation. **(A)** Monotherapy with Esmolol before start of Ivabradine. Time intervals PQ 120 ms, QRS 60 ms, QT 260 ms, QTcB 427 ms. **(B)** After start of Ivabradine, in combination therapy with Esmolol. Time intervals PQ 110 ms, QRS 65 ms, QT 270 ms, QTcB 417 ms. **(C)** Follow up after 10 weeks combination therapy Ivabradine with Metoprolol. Time intervals PQ 110 ms, QRS 60 ms, QT 280 ms, QTcB 404 ms.

The initial 12-lead electrocardiogram (ECG) showed a regular, sustained small-QRS-complex-tachycardia (mean heart-rate 240 beats/min). The tachycardia demonstrated a 1:1 atrio-ventricular conduction (P-P cycle length 250 ms) with a monomorphic P-wave configuration characterized by a superior axis (P-vector of −60°) and sinus tachycardia was therefore not possible. RP interval was significantly longer than PR—interval (i.e., long-RP-tachycardia) and we thought about a permanent orthodromic supraventricular tachycardia first (such as permanent junctional reciprocating tachycardia) (Figure 1) and gave Adenosine intravenously. This did not stop atrial tachycardia and ruled out a reentry-tachycardia dependent on the AV-node. We therefore hypothesized a focal ectopic atrial tachycardia. Infusion of Flecainide (1 mg/kg/30 min) did not affect the atrial rate or atrioventricular conduction. Synchronized electric cardioversion with 0.5 and 1 J/kg was performed due to signs of hemodynamic instability but this just interrupted the tachycardia for seconds. Medical cardioversion and rate control were attempted starting Amiodarone with a bolus. After 30 min the girl developed a second-degree AV-block (type Wenckebach) with ongoing QRS-amplitude alternans, eventually resulting in a bradycardia with 70 beats/min. Amiodarone infusion was stopped and the AV-block resolved. While persisting EAT, Esmolol infusion was started and slowly increased to a maximum rate of 75 mcg/kg/min. This lowered the atrial tachycardia rate to 160 beats/min without affecting the blood pressure. Ivabradine was started with a dose of 0.25 mg (0.025 mg/kg) every 12 h and increased to 0.5 mg (0.05 mg/kg) per dose on the second day. This led to a persistent heart rate reduction to a physiologic level (80–100 beats/min at rest) with alternating periods of restored sinus rhythm and ectopic atrial rhythm. Subsequent ECG recordings showed sinus rhythms with regular AV conduction and no abnormalities of de- or repolarization (Figure 2). No adverse effects attributable to Ivabradine were registered. After stabilization Esmolol infusion was discontinued and the beta-blocker therapy was switched to Metoprolol administered orally (1 mg/kg/d). Holter evaluation before discharge (1 week after admission) showed sinus rhythm with short times of ectopic atrial rhythm and a regular diurnal pattern and heart rate.

**Figure 3:**
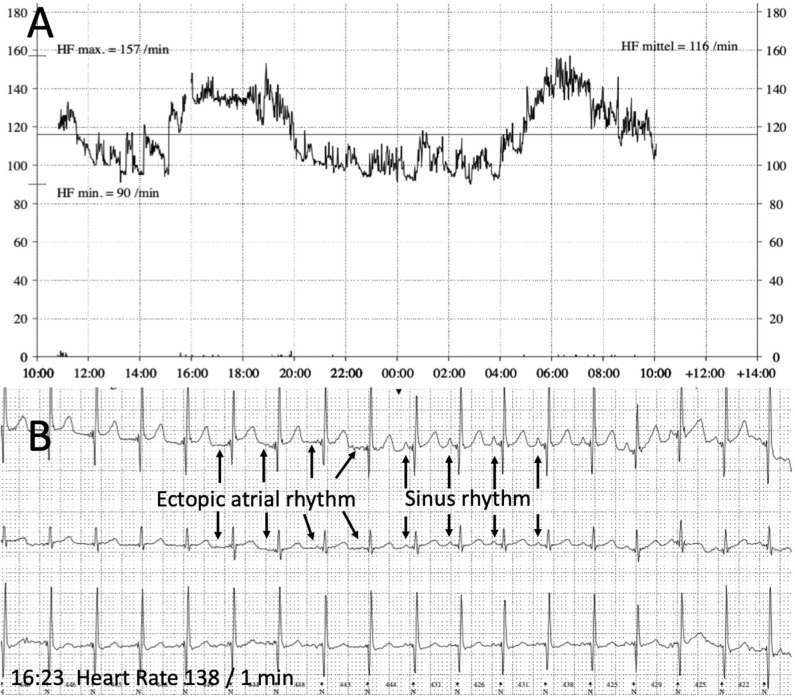
Holter recording (follow up). **(A)** 24 h Heart rate recording showing a physiologic diurnal heart rate spectrum (heart rate maximum 157 beats/min, minimum 90 beats/min, mean 116 beats/min). **(B)** ECG with alternating sinus rhythm and ectopic atrial rhythm during a period with physical activity and accelerated heart rate in the afternoon (4.00–6.00 pm).

In the follow up visits 2 and 10 weeks later echocardiography showed normal left-ventricular function and dimension. The 12-lead-ECG and Holter evaluation showed alternating ectopic atrial and sinus rhythm with a physiologic diurnal heart rate spectrum and a regular response to physical activity (Figure 3). Combination of Metoprolol and Ivabradine did not cause bradycardia and no other side effects attributable to medication were noticed.

## Discussion

We report a case of an 1-year old girl with EAT successfully treated with a combination of beta-blocker and Ivabradine. EAT is a rare cause of supraventricular tachycardia in children (1) due to an ectopic rapid automatic atrial pacemaker (10) and it can lead to a tachycardia induced cardiomyopathy (11). Familiar cases of EAT with autosomal dominant inheritance have been described, but family history was negative in our patient. As the girl presented with supraventricular tachycardia, a moderately reduced contractility and a preceding febrile infection, an acute myocarditis could have been possible. However, there was no further evidence for myocarditis.

Our patient presented with the characteristic ECG features of an EAT showing a persisting atrial tachycardia with a variable rate inappropriate for age. P-wave morphology depends on the atrial origin and was in our case indicative for a origin from the lower left septum or the ostium of the coronary sinus (Figure 1). It usually differs from the P-wave morphology in sinus rhythm although it can be difficult to differentiate atrial origins close to the sinus node. Adenosine can be diagnostic as it induces transient AV-block clearly showing the atrial tachycardia (12).

The clinical course of EAT differs between young children and those 3 years and older (2). Antiarrhythmic medication can be successful in young individuals with a rhythm control rate up to 91% (2) and a spontaneous resolution of ~50% during the first year of life (13). However, a combination therapy of two or more antiarrhythmics is needed in up to 50% of patients with beta-blocker, Amiodarone, Digoxin, Flecainide, and Sotalol being used (13). We used Flecainide as first line agent, which had no effect on tachycardia in the reported case. Amiodarone, as added intravenously, resulted in a bradycardia induced by an AV-block and needed to be discontinued.

As a monotherapy with beta-blockers was not sufficient for rate control we added Ivabradine. Reports on its use for antiarrhythmic therapy in children are rare. Ivabradine however has successfully been used as an addon-therapy in series of drug-refractory infants with congenital JET (4, 5). Just as EAT, congenital JET is caused by enhanced automaticity of cardiac conduction tissue. It seems likely that ivabradine could therefore be useful in patients with EAT too. There are first reports on the efficacy of Ivabradine to control EAT in adult patients as well as in older children and adolescents (6–9). On this basis we started oral Ivabradine in our young patient and restored sinus rhythm within 2 days by a combination therapy with metoprolol. As beta-blocker therapy is established and usually well-tolerated in infants and young children we decided to use Ivabradine not as monotherapy, but in addition to Metoprolol. This combination of Ivabradine and beta-blocker was effective and did not cause bradycardia.

In infants and young children, the anatomic substrate of EAT is different compared to older patients. It has been hypothesized that EAT in small individuals is based on abnormal embryonic cells with enhanced automaticity (14) and is linked with a high risk for complications during catheter ablation due to the low body weight of these children. As spontaneous resolution of the tachycardia is more likely, initially medical treatment is usually preferred. In this context our report adds the second case of an ectopic atrial tachycardia in this age group successfully controlled with Ivabradine to the existing literature. Ivabradine seems to be a promising treatment option in pediatric arrhythmia due to increased automaticity.

No relevant cardiac side-effects attributable to Ivabradine were noticed in our patient during follow-up. This is in concordance with the existing reports supporting Ivabradine as a safe treatment option, but data on its use especially in younger children is very rare. Clinical studies are needed to evaluate its safety and effectiveness systematically compared to standard regimes in the treatment of tachycardia in the pediatric age group.

## Data Availability Statement

The datasets generated for this study are available on request to the corresponding author.

## Ethics Statement

Any identifiable information was removed from the script. Written informed consent for the publication of this case report was obtained from the mother.

## Author Contributions

HM, FH, and SG performed the literature review and wrote the manuscript. MM and SW critically reviewed the manuscript.

## Conflict of Interest

The authors declare that the research was conducted in the absence of any commercial or financial relationships that could be construed as a potential conflict of interest.
